# A Gated Dilated Convolution with Attention Model for Clinical Cloze-Style Reading Comprehension

**DOI:** 10.3390/ijerph17041323

**Published:** 2020-02-19

**Authors:** Bin Wang, Xuejie Zhang, Xiaobing Zhou, Junyi Li

**Affiliations:** School of Information Science and Engineering, Yunnan University, Kunming 650091, China

**Keywords:** clinical medicine, machine reading comprehension, cloze-style, Gated Dilated Convolution, attention mechanism

## Abstract

The machine comprehension research of clinical medicine has great potential value in practical application, but it has not received sufficient attention and many existing models are very time consuming for the cloze-style machine reading comprehension. In this paper, we study the cloze-style machine reading comprehension in the clinical medical field and propose a Gated Dilated Convolution with Attention (GDCA) model, which consists of a gated dilated convolution module and an attention mechanism. Our model has high parallelism and is capable of capturing long-distance dependencies. On the CliCR data set, our model surpasses the present best model on several metrics and obtains state-of-the-art result, and the training speed is 8 times faster than that of the best model.

## 1. Introduction

Machine reading comprehension is a challenging task in natural language processing, and the purpose of this task is to measure the extent to which the machine understands natural language by having the computer read a document and answer its questions [1]. The machine reading comprehension task has made significant progress in the open domain and becomes the research focus of academia and industry. With the development of machine reading comprehension research, many successful models have been proposed. Although the models trained in the general fields can adapt to the new target domain, but the domain mismatch problem usually leads to their performance degradation [2]. Therefore, building new models for specific fields is a challenge.

Due to the lack of large-scale data sets, there are currently no universal systems that can answer the natural questions raised by doctors in clinical reports. In the clinical field, machine reading comprehension tasks are still relatively unexplored [3]. Some research communities began to launch competitions on machine reading comprehension in the clinical field, such as MEDIQA 2019 [4], BIOASQ [5], etc. These tasks have attracted some researchers to carry out various researches, and have played dramatic roles in promoting researches in the clinical medical field [6]. And some related data sets have been proposed, such as CliCR [7], PubMedQA [8], Chimed [2] and emrQA [3] etc. Besides, the clinical field has accumulated extensive experience and knowledge, some of which have been uploaded to PubMed, one of the literature databases in the biomedical field, and has nearly 2 million publications with case types [9,10]. These articles are indexed and account for approximately 7% of all biomedical articles [11]. Clinical case reports can provide valuable, unique, noisy, and underutilized evidences [12]. Often, a case report has only one major finding, which first represents the reason for the report [13]. Therefore, automatic analysis of clinical medical reports by machines will bring great value to future medical research and practical applications.

Currently, clinicians address patient-specific problems by manually browsing or searching for literature and electronic health records. The Question Answering system can simplify this task and bring convenience to medical research. Moreover, in the cloze-style machine reading comprehension task, there is still a lack of research in the field of clinical medicine. Till now, only the CliCR data set has been proposed, and the Stanford Attentive reader(SA) [14] and Gated-Attention Reader (GA) [15] are used as the benchmark models. In the open domain, many models have been proposed, but they are not very suitable for the clinical medical field, and the training time for these models is generally long, which is not conducive to do more research [16]. Therefore, it is particularly essential to propose a cloze-style machine reading comprehension model that is efficient and suitable to the clinical medical field.

**Figure 1:**
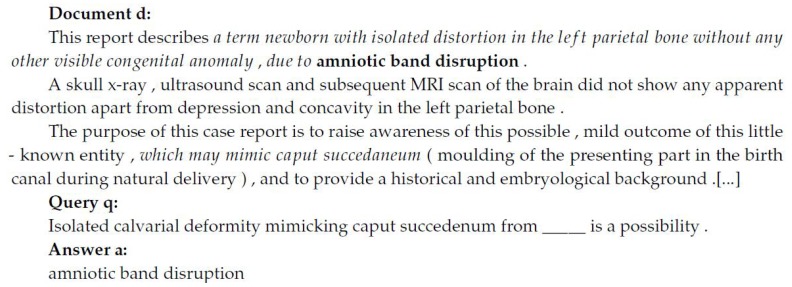
An example from the CliCR data set.

In this paper, we investigate the cloze-style machine reading comprehension on clinical medical data. The data set of this study is CliCR, which uses clinical case reports for a total of nearly 12,000 reports, ranging from 2005 to 2016, and around 100,000 gap-filling queries about these cases. An example from this data set is shown in Figure 1.

The main motivations to design our model are as follows: (1) Most of the existing models are composed of the recurrent model and attention mechanism; (2) The recurrent model is not parallel computing [17], and as the length of the text increases, the amount of computation and time will also increase substantially. To overcome the above-mentioned shortcomings, the structure of our model consists of convolution and attention mechanisms, because the convolution can capture local features, and has high parallelism, which does not increase the time as the length of the text increases. The attention mechanism can capture the interaction information between the document and the query.

## 2. Related Work

The cloze-style machine reading comprehension task can be expressed with a four-tuple form (d,q,a,c), in which *d* is the machine-readable document, *q* is the query corresponding to the document *d*, *a* is the answer to the query *q*, and *c* is the candidate answer pool for the query *q*. Next, we introduce the research development of the cloze-style reading comprehension for the open domain and its current research in the clinical medical field [18,19].

In the open domain, there are already many proposed data sets, such as the CNN/Dailymail data set [20], which is about one million pieces of news data, the Children’s Book Test(CBT) data set [21], which is collected in 108 children’s books [22]. The LAMBADA data set [23] was proposed to expand the language model to solve the problem of discourse. The Who-did-What data set was proposed by Onishi et al. [24], and was only focused on the personal name entity. The CLOTH data set [25] was collected from the English test for Chinese students. Based on these data sets, many models were proposed. For example, the MemNets model [26] has long-term and easy-to-read memory; the EpiReader model [27] can first perform a simple interaction to get a small set of candidate answers, and then use the hypothetical method to reorder and select the final answer; Chen et al. [14] proposed a bilinear matching function in the Stanford Attentive reader(SA) model; Kadlec et al. [28] believed that the correct answer word would appear more times in the document, so the Attention Sum reader was proposed; Cui et al. [29] proposed a new approach(AOA), by adding a new level of attention to the original one to describe the importance of each attention. The Iterative Attentive reader by Sordoni et al. [30] dynamically constructs the correlation between the query, document, and inference states. Dhingra et al. [15] proposed a Gated-Attention(GA) model different from the traditional attention mechanism. Shen et al. [31] proposed a model to dynamically determine the number of rounds of reasoning by reinforcement learning. BiDAF [32] uses a multi-stage and hierarchical process, which makes it possible to capture features of different sizes of the original text. Meanwhile, a bidirectional attention flow mechanism is used to obtain the representation between the relevant question and the original text in the case of without early summarization. Among them, the GA model has obtained state-of-the-art results on many data sets. These models have two characteristics: the recursive module to encode sequential inputs to get sequential information, and the attention mechanism to capture interactive features.

However, for the clinical medical field, there is a lack of research on cloze-style reading comprehension, and there’s only one CliCR data set. The state-of-the-art GA model in the open domain is the best one on this data set. For the CliCR data set, we propose a GDCA model, which exceeds the GA model in multiple evaluation metrics and obtains state-of-the-art result. It is nearly 8 times faster than the GA model in the training time.

## 3. Model

**Figure 2:**
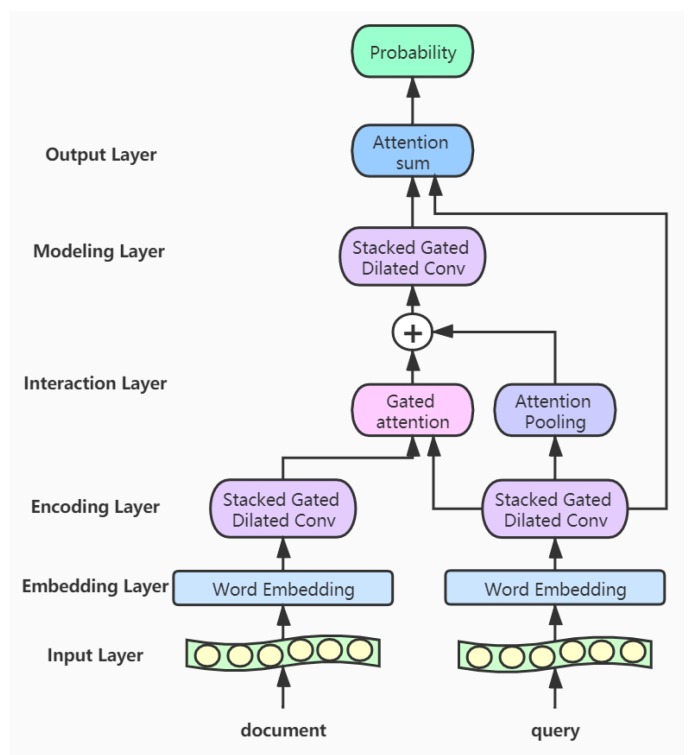
The architecture of Gated Dilated Convolution with Attention model.

In this section, we introduce the proposed GDCA model, which consists of six parts: the input layer, the embedding layer, the encoding layer, the interaction layer, the modeling layer, and the output layer. First, the documents and queries are transformed into high-dimensional word vectors through the embedding layer, respectively. Then the respective features of the documents and queries are extracted through the gated dilated convolution module in the encoding layer [33,34]. In the interaction layer, we use the gated attention mechanism and the attention pooling mechanism to process the features of the documents and queries and obtain a new document representation vectors. Next in the modeling layer, we use stacked gated dilated convolution module to capture the words that are more relevant to the query in the document. We further calculate the relations between words for the document in the modeling layer, and predict the results in the output layer. The structure of our model is shown in Figure 2.

**Figure 3:**
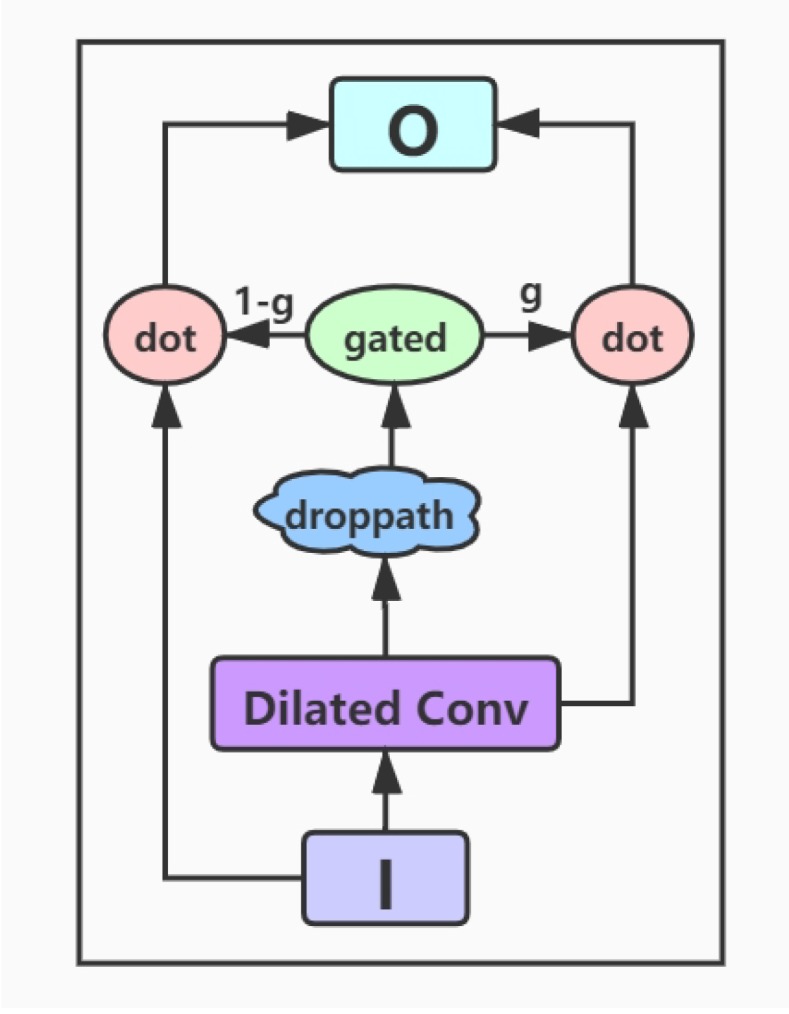
The architecture of Gated Dilated Convolution module.

- **Input layer:** This layer inputs the documents and queries into the model, and a variable-length approach is adopted so that the input text won’t be truncated.
- **Embedding layer:** In this layer, we convert the words of the input texts into word vector representations. The corpus for the word embedding is only from the CliCR training set and is learned by GloVe [35].
- **Encoding layer:** The encoding layer is a stack of gated dilated convolution modules, whose structure is similar to the Gated Linear Unit(GLU) [34,36]. The GLU structure is proposed by Facebook, and its advantage is that it hardly has to worry about the gradient disappearing because part of it is without any activation function. And dilated convolution can capture farther distances than conventional convolutions without causing any increase in parameters [37]. Once the convolution kernel and the step size are determined, the receptive field of the conventional convolution has a linear relationship with the number of layers of convolution, and the dilated convolution is an exponential relationship. The structure of the Gated Dilated Convolution module(GDConv) is shown in Figure 3.
  Assuming the document sequence $D=[d_{1},d_{2},\ldots ,d_{k}]$, where *k* is the number of sentences in the document, the query sequence $Q=[q_{1},q_{2},\ldots ,q_{n}]$, where *n* is the number of sentences in the query. We input them into the dilated convolution layer and get a single output element $D_{c}$ and $Q_{c}$, respectively, with the dimension of 2d:
  (1) $$\begin{array}{c} D_{c}=DilatedConv\left(D\right),\\ Q_{c}=DilatedConv\left(Q\right). \end{array}$$
  We divide the above outputs into two equal parts *X* and *Y*, both with the dimension *d*, which can be expressed as $D_{c}=\left[X_{D}\;Y_{D}\right]$, $Q_{c}=\left[X_{Q}\;Y_{Q}\right]$. Then, we use the activation function sigmoid on *Y* to control which inputs *X* of the current context are relevant to, and perform the element-wise multiplicative operation with *X*. The formula is as follows:
  (2) $$\begin{array}{c} D_{g}=X_{D}⊗f\left(Y_{D}\right),\\ Q_{g}=X_{Q}⊗f\left(Y_{Q}\right). \end{array}$$
  In order to solve the gradient disappearance problem and make the information transmit through multiple channels, the residual structure is used here, and the input sequence is also added. The formula is as follows:
  (3) $$\begin{array}{c} D_{g}=D⊗\left(1-f\left(Y_{D}\right)\right)+X_{D}⊗f\left(Y_{D}\right),\\ Q_{g}=Q⊗\left(1-f\left(Y_{Q}\right)\right)+X_{Q}⊗f\left(Y_{Q}\right). \end{array}$$
  Then we use a droppath-like regularization method to make the model more robust:
  (4) $$\begin{array}{c} D_{g}=D⊗\left(1-f\left(Y_{D}⊗\left(1+\varepsilon \right)\right)\right)+X_{D}⊗f\left(Y_{D}⊗\left(1+\varepsilon \right)\right),\\ Q_{g}=Q⊗\left(1-f\left(Y_{Q}⊗\left(1+\varepsilon \right)\right)\right)+X_{Q}⊗f\left(Y_{Q}⊗\left(1+\varepsilon \right)\right). \end{array}$$
- **Interaction layer:** Here we use the gated attention module proposed by Dhingra et al. [15], which obtains *q* by the soft attention, and then performs the element-wise multiplicative operation with the document representation vector *d*. The formula is as follows:
  (5) $$\begin{array}{c} x_{i}=d_{i}⊗\left(Q_{g}softmax\left(Q_{g}^{T}d_{i}\right)\right). \end{array}$$
  Here we use the additive attention mechanism instead of simple pooling to complete the integration of the sequence information [38], namely, to encode the vector sequence of the query into a total query vector. Its formula is as follows:
  (6) $$\begin{array}{cc} \alpha_{i} & =softmax(\beta^{T}f\left(Wq_{i}\right)),\\ \tilde{q} & =\sum_{i=1}^{n}\alpha_{i}q_{i}. \end{array}$$
  We concatenate the total query vector into the document representation $D_{g}$ and get the new document representation vector $D_{h}$
  (7) $$\begin{array}{c} D_{h}=x_{i}⊕\tilde{q}. \end{array}$$
- **Modeling layer:** The input to this layer is $D_{h}$, which encodes the new representation of document words. Unlike the coding layer, since the representation of these words contain information about query’s integration, it can capture the words that are more relevant to the query in the document. We use five layers of Gated Dilated convolution(GDConv). Moreover, the dilated_rate of each layer is almost doubled, with the aim of establishing a farther relationship between words
  (8) $$\begin{array}{c} D_{f}=DilatedConv\left(D_{h}\right). \end{array}$$
- **Output layer:** In this layer, we calculate the inner product of the resulting document representation $D_{f}$ and the query representation $Q_{g}$ and pass them through a softmax layer as the normalizing weights
  (9) $$\begin{array}{c} s=softmax(Q_{g}^{T}D_{f}). \end{array}$$
  The vector *s* represents the probability of a word in the document. Then we integrate the probability of all the same words in the document for candidate set *C*. And this operation is the same as that in the AS model [28]:
  (10) $$\begin{array}{c} Pr\left(c|d,q\right)\propto \sum_{i\in (c,d)}s_{i}, \end{array}$$
  where (c,d) indicates that the set of candidate *c* appears in document *d*.
  Finally, we calculate the candidate answer *c* with the highest probability as the final predicted answer:
  (11) $$\begin{array}{c} \hat{a}=argmax_{c\in C}Pr\left(c|d,q\right). \end{array}$$

## 4. Experiments and Results Analysis

### 4.1. Data Set Description

Our research is based on the CliCR data set, which is sourced from the BMJ Case Reports. About 100,000 queries in this data set are answered by 50,000 distinct entities. For each entity, a Concept Unique Identifier (CUI) is also used to link it to UMLSR Metathesaurus. The maximum doc length of the document in the data set exceeds 3000, and the average doc length is 1466. So the model must have long-term dependencies. The numbers of queries in the training set, validation set, and test set are 91,344, 3691, and 7184, respectively. Since no candidate answers are provided in the data set, we limit the candidate to the collection of entities in the paragraph. The specific data set details are shown in Table 1.

### 4.2. Experiment Setting

For the gated convolution module in our model, all convolutions have a window size of 3 and the interference term ε of 0.1. In the coding layer, we use two layers of DGConv for the document, and their dilated_rate are 1 and 2, respectively; and the query uses three layers of DGConv, the dilated_rates are 1, 2 and 1, respectively. In the modeling layer, we use five layers of DGConv for the document, their dilated_rates are 1, 2, 5, 9, and 17, respectively. This is to ensure that the dilated_rate in the stack convolution has no common divisor greater than 1, to guarantee the consistency of the information. In the interaction layer, we add a dropout layer, and the drop_rate is 0.4, to prevent the model from overfitting and improve the performance of the model [39]. The reason for the design of the hyperparameters is the optimal solution found by the grid search algorithm. In addition, for the dilated convolution, if the $dilated_{r}ate$ of the previous layer and the subsequent layer have the same common divisor, the continuity of information will be lost.

During the training stage, we set the batch size to 40 and the epoch to 3. The loss function of this model is cross-entropy, and the optimizer is Adam [40].

**Word embedding.** Since many words from the data set are not in the existing pre-trained word embedding and there are a large number of unknown words. So we use the CliCR training set as a corpus to build word embedding by GloVe. We set the dimension of the word embedding to 100, window_size to 15, vocab_min_count to 0, max_iter to 100.

### 4.3. Results Analysis

**Metrics.** Our main evaluation metrics are exact match(EM) and F1 score, which are the two popular evaluation metrics in machine understanding. For the EM, the predicted answers and the ground truth answers must be exactly matched. The F1 scores is a common indicator in machine reading comprehension tasks, in which candidate answers and reference answers are both considered token bags, and the final predicted results can be divided into true positives (TP), false positives (FP), true negatives (TN) and false negatives (FN). Then precision(P) and recall(R) can be calculated by the following formula

(12) $$\begin{array}{c} P=\frac{TP}{TP+FP},\\ R=\frac{TP}{TP+FN}. \end{array}$$

Then, the formula for the F1 score is as follows

(13) $$\begin{array}{c} F1=\frac{2PR}{P+R}. \end{array}$$

In addition, the CliCR data set also introduces two additional metrics BLEU-2(B-2) and BLEU-4(B-4), because medical entities may have potential large lexical and word order variation. The BLEU score not only evaluates the similarity between the candidate answer and the real answer, but also tests the readability of the candidate, which is calculated as follows:

(14) $$\begin{array}{c} P_{n}\left(C,A\right)=\frac{\sum_{i}\sum_{j}min\left(h_{j}\left(c_{i}\right),max\left(h_{j}\left(a_{i}\right)\right)\right)}{\sum_{i}\sum_{j}h_{j}\left(c_{i}\right)},\\ BLEU=BP.exp(\sum_{N}^{n=1}w_{n}logP_{n}). \end{array}$$

where $h_{j}\left(c_{i}\right)$ calculates the number of j-th n-grams appearing in candidate answer $c_{i}$; similarly, $h_{j}\left(a_{i}\right)$ represents the number of occurrences of the n-gram in gold answer $r_{i}$. BP is a penalty term, and when the length of candidate answer is greater than real answer, BP=1, otherwise $BP=e^{1-\frac{n_{a}}{n_{c}}}$, $n_{a}$ and $n_{c}$ are the length of answer and candidate answer, respectively. *N* means n-grams up to length *N*, $w_{n}=\frac{1}{N}$.

**Results.** Our results are shown in Table 2. Our model has reached the highest in all of the above metrics. Compared with the previous best GA-Anonym model, our model is 1.2% higher for the EM, 2.1% higher for the F1 score, and the other two metrics B-2 and B-4 are also improved by 0.01. Compared with GA-NoEnt, our model has an increase of 10.8% for the EM and a 1.4% improvement for the F1 score. There are also significant improvements in the other two additional metrics B-2 and B-4, which are 0.08 and 0.10, respectively.

The performances of the baselines rand-entity and maxfreq-entity presented in [7] are very poor because a random entity and the most frequent entity in the passage are used as answers, respectively. The lang-model method performs poor because it is based on queries only, without reading the document, it is difficult to provide accurate answers. The sim-entity method is a traditional one and is inferior to the neural network model because the method only compares the similarity of the words between the query and the document, and does not further infer the words from the document. The SA model is an end-to-end neural network, which learns semantic matches involving paraphrasing or lexical variation between the two sentences, but the performance is still not satisfying. The GA model is more effective than all the previous models, but we observe that the model can only infer the relationship between the closer words in the document during the reasoning process, and the possible relations between the more distant words have not been obtained. Therefore, our proposed GDCA model outperforms the previous models with the gated dilated convolution module to make long-term dependencies between words and attention pooling mechanism to integrate the features of the query into a vector to assist the document’s further reasoning.

**Speedup over GA model.** We compare the GA model on the training time with our model under the same hardware. The comparison results are shown in Table 3. In our experiments, we use a GTX1080 Ti GPU, both models are based on the Keras framework with the Theano as the backend, and the batch size is 40.

It is not difficult to observe from the above table that our model is 8 times faster than the GA model during the training stage, which proves the high efficiency of our model.

### 4.4. Ablation Study for Model Components

In the following, we perform ablation studies on the components of our model on the CliCR data set. We experiment with the four components of embedding, dilated convolution, attention pooling, and gated attention. And we only use the two metrics of EM and F1 score for ablation study.

**The Influence of Word Embedding.** The comparison between the pre-trained word embedding [15] and the word embedding based on the CliCR training set is shown in Table 4. For the pre-trained word embedding, nearly 55% of the words are unknown, so they are randomly initialized. As can be seen from the table, these unknown words adversely affect the performance of the model.

**The Influence of Dilated Convolution.** It is not enough to establish a relationship between words and the surrounding words. Therefore, we use the dilated convolution to make a long-term dependency similar to RNN. This approach is demonstrated to be effective from experiments, and the results of the comparison between convolution and dilated convolution in the model are shown in Table 5.

**The Influence of Attention Pooling.** The information of the query is integrated into the words in the document, so that the model can better infer the answer and improve the performance of the model. The results of the model with and without attention pooling are shown in Table 6.

**The Influence of Gated Attention.** The interaction between the document and the query is critical, it can measure the importance of the words related to the problem in the document. From Table 7, we can see the importance of the gated attention.

## 5. Conclusions

In this paper, we present a DGCA model, which has high parallelism and is nearly 8 times faster than the previous best GA model. It saves a lot of training time and can train more data than other models with the same time. This brings great convenience to practical applications and scientific research. On the CliCR data set, our model achieves the highest score on several metrics and obtains state-of-the-art result. Because the convolution has no way to get the sequential information well, we will try to solve this problem in our future work, so that the model can improve performance without slowing down the training speed. In addition, we’ll study how to handle natural language in connection with reinforcement learning to further improve our model’s performance in the future.

## Figures and Tables

**Table 1:** The data set details for CliCR.

Category	Number
Cases	11,846
Queries in train/dev/test	91,344/6391/7184
Tokens in documents	16,544,217
Distinct answers	56,093
Distinct answers(extended)	288,211
Entity types in documents	591,960

**Table 2:** Results on test set for CliCR (EM and F1 scores are in percentage) [7].

Model	EM	F1	B-2	B-4
human−expert	35	53.7	0.46	0.23
human−novice	31	45.1	0.43	0.24
rand-entity	1.4	5.1	0.03	0.01
maxfreq-entity	8.5	12.6	0.10	0.05
lang-model	2.1	3.5	0.00	0.00
sim-entity	20.8	29.4	0.22	0.15
SA-Anonym	19.6	27.2	0.22	0.16
SA-Ent	6.1	11.4	0.07	0.05
GA-Anonym	24.5	33.2	0.28	0.20
GA-Ent	22.2	30.2	0.25	0.18
GA-NoEnt	14.9	33.9	0.21	0.11
**Our model**	**25.7**	**35.3**	**0.29**	**0.21**

**Table 3:** The training time comparison between GA Reader model and our model on CliCR data set.

Model	Time per epoch
GA Reader	6 h 40 min
our model	50 min

**Table 4:** The comparison between the CliCR training set embedding and the pre-trained embedding.

Embedding	EM	F1
Pre-trained	25.4	34.7
CliCR training set	25.7	35.3

**Table 5:** The comparison between the convolution and dilated convolution.

Method	EM	F1
Convolution	24.6	34.0
Dilated convolution	25.7	35.3

Note: the number of Convolution layers is the same as that of Dilated Convolution layers.

**Table 6:** The comparison between the model with and without attention pooling.

Method	EM	F1
Attention pooling/o	25.0	34.9
Attention pooling/w	25.7	35.3

**Table 7:** The comparison between the model with and without gated attention.

Method	EM	F1
Gated attention/o	19.2	27.3
Gated attention/w	25.7	35.3
